# *In Vivo* Quantification of Inflammation in Experimental Autoimmune Encephalomyelitis Rats Using Fluorine-19 Magnetic Resonance Imaging Reveals Immune Cell Recruitment outside the Nervous System

**DOI:** 10.1371/journal.pone.0140238

**Published:** 2015-10-20

**Authors:** Jia Zhong, Kazim Narsinh, Penelope A. Morel, Hongyan Xu, Eric T. Ahrens

**Affiliations:** 1 Department of Radiology, University of California San Diego, School of Medicine, La Jolla, California, United States of America; 2 Department of Immunology, University of Pittsburgh School of Medicine, Pittsburgh, Pennsylvania, United States of America; Johns Hopkins University, UNITED STATES

## Abstract

Progress in identifying new therapies for multiple sclerosis (MS) can be accelerated by using imaging biomarkers of disease progression or abatement in model systems. In this study, we evaluate the ability to noninvasively image and quantitate disease pathology using emerging “hot-spot” ^19^F MRI methods in an experimental autoimmune encephalomyelitis (EAE) rat, a model of MS. Rats with clinical symptoms of EAE were compared to control rats without EAE, as well as to EAE rats that received daily prophylactic treatments with cyclophosphamide. Perfluorocarbon (PFC) nanoemulsion was injected intravenously, which labels predominately monocytes and macrophages *in situ*. Analysis of the spin-density weighted ^19^F MRI data enabled quantification of the apparent macrophage burden in the central nervous system and other tissues. The *in vivo* MRI results were confirmed by extremely high-resolution ^19^F/^1^H magnetic resonance microscopy in excised tissue samples and histopathologic analyses. Additionally, ^19^F nuclear magnetic resonance spectroscopy of intact tissue samples was used to assay the PFC biodistribution in EAE and control rats. *In vivo* hot-spot ^19^F signals were detected predominantly in the EAE spinal cord, consistent with the presence of inflammatory infiltrates. Surprising, prominent ^19^F hot-spots were observed in bone-marrow cavities adjacent to spinal cord lesions; these were not observed in control animals. Quantitative evaluation of cohorts receiving cyclophosphamide treatment displayed significant reduction in ^19^F signal within the spinal cord and bone marrow of EAE rats. Overall, ^19^F MRI can be used to quantitatively monitored EAE disease burden, discover unexpected sites of inflammatory activity, and may serve as a sensitive biomarker for the discovery and preclinical assessment of novel MS therapeutic interventions.

## Introduction

Multiple sclerosis (MS) is an autoimmune inflammatory and demyelinating disease of the central nervous system (CNS) that affects millions of people worldwide. The progressive form of MS results in severe neurologic disability due to an accumulation of physical and cognitive impairments [1, 2]. Treatment of progressive MS is especially challenging because of the chronicity of the disease process. The complex cascade of MS lesion development involves blood-brain barrier (BBB) breakdown, infiltration of inflammatory cells, demyelination, axonal damage, and microglial activation [3]. Experimental autoimmune encephalomyelitis (EAE), a family of disease models with key immunopathological features mimicking MS, has been widely used to evaluate potential therapies in their preclinical stage [4, 5]. Both MS and EAE are T cell-mediated diseases, where perivascular and parenchymal infiltrates target myelin antigens in the CNS. Infiltrating tissue macrophages have been reported as crucial effectors of EAE disease activation and progression [6–9]. The role of macrophages in EAE includes phagocytosis, antigen presentation, secretion of and response to proinflammatory cytokines, production of nitric oxide (NO) and superoxide radicals, and impairment of BBB and oligodendrocyte function [10].

Quantitative monitoring of inflammation in EAE could serve as a diagnostic for disease progression and aid in the development and evaluation of novel treatments. Traditional methods for assaying inflammation, such as histological staining and flow cytometry, are laborious and necessitate tissue destruction, thereby precluding longitudinal assessment. *In vivo* imaging techniques can overcome this bottleneck by noninvasively and quantitatively assessing inflammation repeatedly over time. MRI is the modality of choice to noninvasively examine disease severity in MS patients and is often used with Gd-based contrast agents to elucidate active BBB breaches. Preclinical and clinical MRI studies have utilized iron-oxide nanoparticle imaging agents; following intravenous injection, these agents label tissue macrophages to generate hypointense regions in T_2_*-weighted images indicative of inflammatory infiltrates [11–16]. However, these infiltrates represented by T_2_*-weighted signal loss are often indistinguishable from intrinsic or disease pathogenesis contrast sources. Also, T_2_* signal loss is not linearly proportional to the number of infiltrating cells, thereby confounding quantitative image analysis [17].

Prior work has demonstrated *in situ* macrophage labeling and tracking methods using fluorine-19 (^19^F) MRI [18–30], including early work in an EAE rat model [27]. In this approach, perfluorocarbon (PFC) nanoemulsion is administered intravenously to a subject, and the nanoemulsion droplets are taken up *in situ* by cells of the reticuloendothelial system (RES), particularly circulating phagocytes in the blood such as monocytes, macrophages, neutrophils, and DCs [31]. When labeled cells accumulate in sufficient amounts at sites of inflammation, they become detectable by spin-density weighted ^19^F MRI. The ^19^F MRI detects the intracellular PFC tracer agent, thereby enabling “hot-spot” tracking of inflammatory cells with high specificity and no background [32]. Macrophages are the dominant contribution to MRI-apparent ^19^F hot-spots. In addition, one can take advantage of the direct, linear correlation between ^19^F signal and macrophage cell number to quantitate the macrophage burden directly from the *in vivo* images [19, 22].

In this study, we explore the use of PFC imaging probes and ^19^F MRI to quantitatively assay the inflammatory burden in EAE rats, including diseased subjects treated prophylactically with an anti-inflammatory agent. We quantify ^19^F signal and inflammatory burden *in vivo* in the spinal column, with and without cyclophosphamide treatment. Additionally, ^19^F NMR analysis of panels of excised, intact tissues and organs was also used to examine the overall PFC biodistribution in the rat model. Our results suggest that *in situ* PFC macrophage labeling and MRI is a non-invasive alternative to clinical symptom scoring and histology for objective disease assessment.

## Materials and Methods

### EAE rat model

Experiments were carried out at, and in accordance with the guidelines provided by the Carnegie Mellon University Institutional Animal Care and Use Committee (IACUC), which approved this study (authorization number AS13-03), and the National Institute of Health Guide for the Care and Use of Laboratory Animals. Anesthesia and euthanasia were by isoflurane and CO2 gases, respectively.

The EAE rat model was generated using previously reported methods [22, 33]. For *in vivo* MRI studies, three experimental groups were prepared. In Group 1, N = 5 adult female dark agouti (DA) rats (Harlan, Indianapolis, IN), 11 weeks old, were inoculated (Day 0) with a single subcutaneous injection at the tail base containing homogenizing DA rat spinal cord (100 mg/rat) with 100 μl of incomplete Freund’s adjuvant (IFA; Difco, Detroit, MI) and 2 mg of Mycobacterium tuberculosis. After disease induction, rats in all groups were monitored daily for body weight and clinical symptoms of EAE. Symptoms were scored according to the convention: 0 = normal; 1 = limp tail; 2 = paraparesis with a clumsy gait; 3 = hind-limb paralysis; 4 = hind- and forelimb paralysis; 5 = moribund. We note that no rats progressed to stage 4 or 5. In Group 2 (control), N = 5 rats were inoculated as above, with IFA and mycobacterium tuberculosis, but without the spinal cord homogenate. In Group 3 (therapeutic), N = 5 rats received the same EAE-inducing inoculation as Group 1, but also received intraperitoneal (i.p.) cyclophosphamide (Baxter, Deerfield, IL) at 20 mg/kg body weight and 500 mg/25 ml every five days starting from Day 0.

For *ex vivo* NMR and flow cytometry studies of tissue samples, additional groups were prepared. Groups 4 and 5 were EAE (N = 4) and control (N = 5) rats, respectively, and were prepared identically to Groups 1 and 2, except were used exclusively for NMR analysis of fixed tissues.

In Groups 1 and 4, upon the first signs that an animal reached clinical stage 2 (typically Day 12–14), rats received a single intravenous (i.v.) injection (1 mL) of PFC nanoemulsion (VS-1000H; Celsense, Inc., PA) via the tail vein. The nanoemulsion contains 30% (v/v) perfluoropolyether with a mean droplet size of 145 nm. VS1000H has a T_1_/T_2_ of approximately 500/350 ms at 7 T. The PFC nanoemulsion was formulated for preclinical use only. The total amount of fluorine administered was ~5.0×10^21^ atoms. In Groups 2, 3, and 5, rats were administered PFC at Day 14, as disease progression was not present.

For flow cytometry analyses, we prepared Groups 6 and 7 (N = 2, each) of EAE and control rats, respectively, where the PFC nanoemulsion was rendered fluorescent prior to injection to aid in flow cytometry analysis. We performed a premix step of the PFC nanoemulsion with a lipophilic dialkylcarbocyanine (DiI, V22885, Molecular Probes, Inc., Eugene, OR) fluorophore [22, 34]. The fluorescent inoculant (PFC-DiI) was prepared by mixing 5 mL nanoemulsion with a 2 μl/ml DiI stock solution prepared according to manufacturer's instructions. After incubating the nanoemulsion with DiI for 20 minutes at room temperature under gentile agitation, the DiI molecules became associated with the PFC nanoemulsion droplets. The PFC-DiI nanoemulsion was then injected into Groups 6 and 7 as above.

### *In vivo* ^19^F MRI

Two days after PFC injection, MRI scans were acquired in Groups 1–3. During MRI, animals were anesthetized with 2% isoflurane in 67% O_2_ and 33% N_2_O, placed on a mechanical ventilator (Harvard Apparatus, Holloston, MA), and core temperature was maintained at 37°C using a heated pad. ^19^F/^1^H MRI data were acquired using a 7 T Bruker BioSpec scanner (Billerica, MA). A birdcage volume coil was used that could be tuned to either ^19^F or ^1^H. For each rat, two parasagittal ^19^F spin density-weighted data sets using a ^19^F RARE (Rapid Acquisition with Refocused Echoes) sequence were acquired sequentially to cover the entire spinal cord and hindbrain, where the rat was repositioned between scans. The scan parameters were TR/TE = 1,000/12 ms, RARE factor = 8, number of averages (NA) = 800, field of view (FOV) = 4.5 × 9.0 cm^2^, resolution = 0.7 × 1.4 × 2 mm^3^. To provide anatomical context to the ^19^F slices, T_2_-weighted ^1^H images were acquired using a spin-echo sequence. The ^1^H imaging parameters were TR/TE = 1,000/11 ms, NA = 4, and resolution = 0.23 × 0.23 × 2 mm^3^. A reference capillary with diluted PFC nanoemulsion (5% v/v) in 1% agarose were placed above the animal’s torso, with one near the lungs and the other by the animal’s kidney. The ^19^F/^1^H MRI images were fused and analyzed using Voxel Tracker software (Celsense, Inc., Pittsburgh, PA). For display purposes, the ^19^F was rendered in pseudo-color (“hot-iron”) scale and a threshold mask of SNR = 2.5 was then applied to the images, which removed 99.3% of all voxels containing only noise. In slices, ^19^F regions of interest (ROIs) were manually outlined aided by anatomical guidance from the high resolution ^1^H images. ROIs of the ^19^F reference capillary and background noise were also delineated. The *in vivo* ^19^F signal in ROIs was calculated using Voxel Tracker, as previously reported [19]. The external ^19^F capillary was used as an absolute ^19^F reference, and quantitative analysis yielded the apparent number of fluorine atoms within ROIs. For ^19^F image quantification, full-dynamic range data without thresholds were analyzed.

### *Ex vivo* ^19^F MRI

After *in vivo* MRI experiments, animals were euthanized. The intact vertebrae and spinal cord were excised and fixed in 4% paraformaldehyde (PFA) for 24 hours. Thoracic vertebrae 5–7 were imaged using an 11.7 T Bruker micro-imaging system with a ^19^F/^1^H volume coil. For ^19^F, RARE parameters were TR/TE = 900/12 ms, RARE factor = 8, NA = 2,400, field of view (FOV) = 13.6 × 13.6 × 10.2 cm^3^, and resolution = 0.2 × 0.2 × 0.4 mm^3^. For ^1^H, co-registered, high-resolution T_2_-weighted RARE images were acquired with TR/TE = 800/11 ms, RARE factor = 8, NA = 36, and resolution = 0.05 × 0.05 × 0.1 mm^3^.

### *Ex vivo* ^19^F nuclear magnetic resonance (NMR) of excised tissues

NMR analyses were used to assay ^19^F content in the fixed spinal cord segments and organs from Groups 4 and 5. Two days after PFC injection, these were euthanized by transcardial perfusion with phosphate buffered saline (PBS) followed by 4% PFA. The vertebral columns, with intact spinal cords, and brains were necropsied, as were other intact internal organs and tissues (lymph nodes, spleen, kidney, thymus, heart, lung, liver, femur, brain, salivary glands). The spinal cord, except the sacral region, was partitioned at intervertebral discs into 15 segments, with each segment containing 1 to 3 vertebrae. The spinal cord was dissected and weighed, and the vertebral bone and spinal nerves discarded. The intact brain stem and cerebellum was also dissected from the excised brain and weighed. Intact tissues specimens were placed at the bottom of 10 mm NMR tubes (Wilman-Labglass, Vineland, NJ). The samples had a flame-sealed 1 mm diameter glass capillary tube (Kimble Kontes, Vineland, NJ) containing 10 μL of 2% (v/v) trifluoroacetic acid (TFA) placed inside the NMR tube next to the tissue sample; the total fluorine content of the TFA reference (*F*_*r*_) was 4.9 × 10^18^ atoms. One-dimensional ^19^F NMR spectra were acquired at 470 MHz using a Bruker spectrometer with a repetition time of 5 seconds and 16 averages. Two distinct peaks were observed at approximately -76 and -91.5 ppm for TFA and PFC, respectively. The total fluorine content of a sample was calculated from the integral of the peaks for the PFC (*I*_*s*_) and TFA (*I*_*r*_), as previously described in [22]. Afterwards, the fluorine content was normalized to the sample weight (*W*_*s*_), yielding an inflammation index (*I*_*inf*_ *)* with units of ^19^F atoms/gram of tissue. The inflammation index is defined as:

$$I_{inf}=I_{s}F_{r}/\left(I_{r}W_{s}\right)$$ (1)

### Histology

After *ex vivo* MRI, histology sections were prepared from the fixed vertebrae at thoracic levels 5–7 from Groups 1–2. Tissues were embedded in paraffin, cut into 6 μm-thick transverse sections, and stained with hematoxylin and eosin (H&E).

### Flow cytometry

To confirm the cell populations labeled by PFC, bone marrow was extracted from the femur and vertebral bodies of EAE and control rats that had received PFC-DiI (Groups 6 and 7, respectively). Single cell suspensions were generated, and the cells were stained for expression of CD45, CD11b/c and Ki67. The cells were analyzed on an LSR II (BD Biosciences, San Jose, CA) flow cytometer. The results were analyzed using FlowJo software (FlowJo LLC, Ashland OR).

### Statistics

All measurements are presented as mean ± standard error of the mean (SEM). When comparing two groups of animals, two-tailed T-tests with unequal variances were performed. Multiple groups were compared using one way analysis of variance (ANOVA). P-values < 0.05 were assigned statistical significance.

## Results

**Fig 1:**
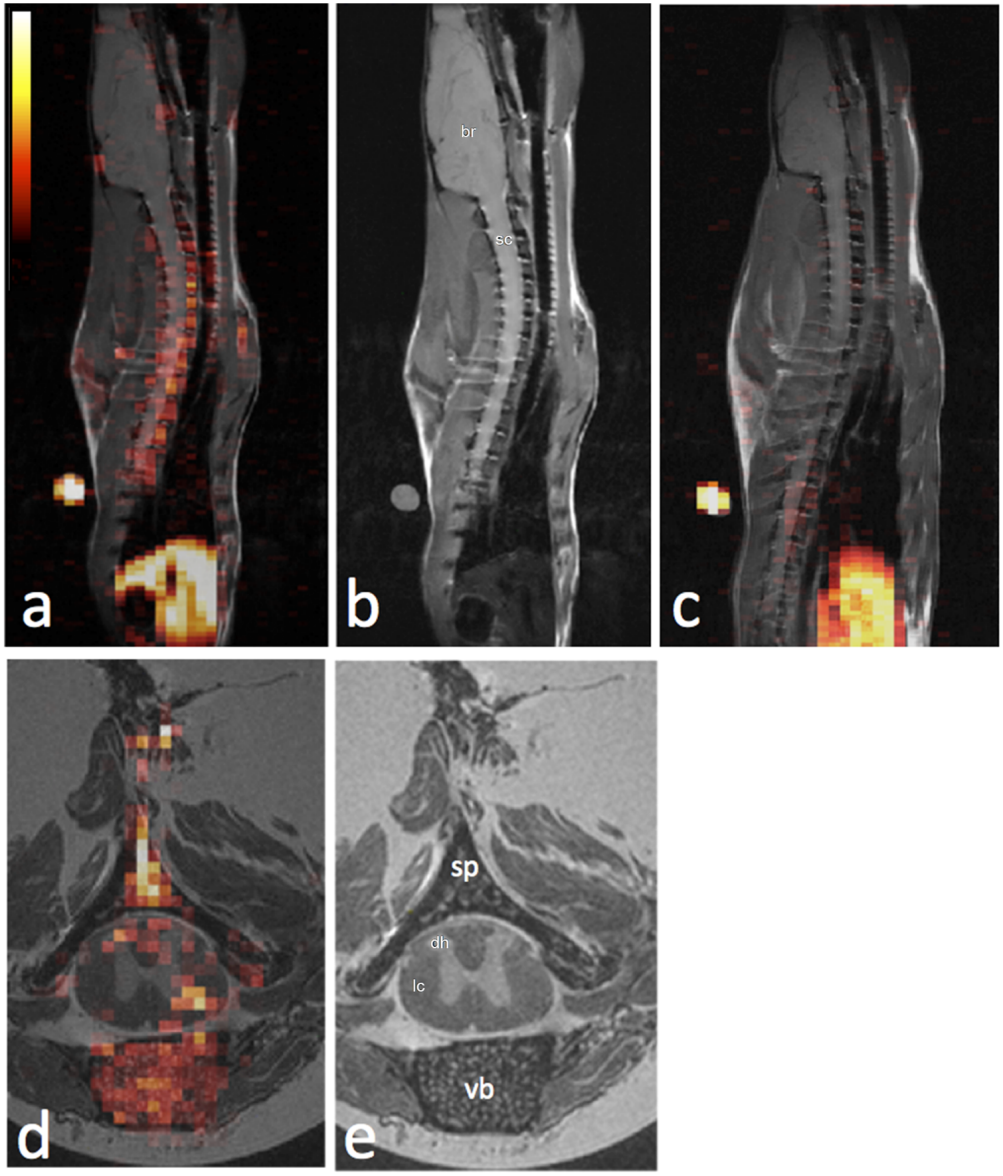
*In vivo* and *ex vivo* ^19^F/^1^H MRI of EAE rat shows ^19^F signal arising from the spinal cord and adjacent vertebral bone marrow, indicating accumulation of inflammatory phagocytes. (**a**) *In vivo* ^19^F/^1^H fused sagittal image of an EAE rat spine demonstrating a long segment of robust ^19^F signal in the spinal cord and vertebrae. ^19^F signal is also appreciated in the RES inferiorly. A “hot iron” pseudocolor calibration scale is displayed in the top right. (**b**) The ^1^H image component of the fused image shown in (**a**) is shown for anatomic detail. (**c**) *In vivo* ^19^F/^1^H fused sagittal image of a control, wild-type rat, demonstrating no significant ^19^F signal in the spine. ^19^F signal is again appreciated in the RES inferiorly. (**d**) *Ex vivo* ^19^F/^1^H fused axial image at the level of thoracic vertebra 6 of an EAE rat demonstrates ^19^F signal indicated inflammatory loci predominantly in the vertebral bone marrow and in the dorsal and lateral columns of the white matter of the spinal cord. Labels are br = brain, sc = spinal cord, vb = vertebral body, lc = lateral column, dh = dorsal horn. (**e**) The ^1^H image component of the fused image shown in (**d**) is shown for anatomic detail.

We employ *in situ* PFC labeling and ^19^F MRI to image inflammation in an EAE model and can quantitatively assess the effects of immunomodulatory treatments *in vivo*. Moreover, we observe significant PFC uptake in vertebral bone marrow cavities adjacent to the spinal cord EAE lesions. Fig 1a displays a typical parasagittal ^19^F/^1^H composite image in an EAE rat (Group 1), with ^19^F rendered in hot-iron pseudocolor and ^1^H in grayscale (Fig 1b). Substantial ^19^F signals are observed predominately in the vertebral column (Fig 1a), compared to control rat from Group 2 (Fig 1c). The liver also displays ^19^F signal, as the RES is the blood clearance pathway of the PFC (Fig 1a and 1c). High-resolution *ex vivo* ^19^F/^1^H MRI images support the *in vivo* findings; substantial PFC is observed in the dorsal and lateral aspects of the spinal cord in EAE rats (Fig 1d and 1e). Additionally, marked ^19^F signal was detected in the bone marrow of the vertebral bodies, laminae, and spinous processes adjacent to the white matter lesions (Fig 1d). Previously it has been shown that the ^19^F signal intensity following i.v. PFC infusion arises predominately from the presence of labeled macrophages and correlates linearly with macrophage burden [19, 22]. Three-dimensional renderings of these high-resolution ^19^F/^1^H data in movie format are provided online in Supporting Information (S1–S3 Movies).

**Fig 2:**
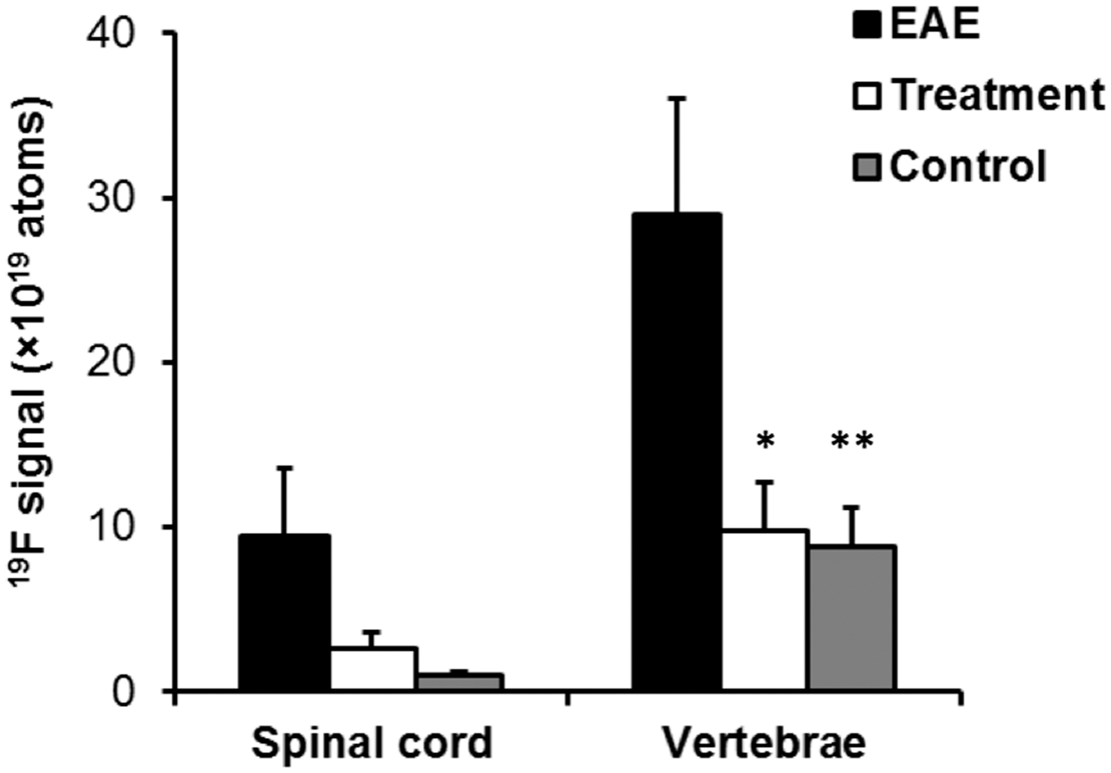
*In vivo* quantification of inflammation load in the spinal cord and vertebral bone marrow in EAE rats. ^19^F signal in the spinal cord and vertebral bone marrow is significantly increased in EAE rats compared to wild-type rats (black vs. grey bars; p < 0.05 indicated by *). ^19^F signal in the spinal cord and vertebral bone marrow of EAE rats is significantly decreased by prophylactic treatment with cyclophosphamide (black vs. white bars; p < 0.05 indicated by **). The apparent number of fluorine atoms was measured by integrating the signal in ROIs and normalizing the results to the signal in a reference capillary containing a known concentration of fluorine, as discussed in Methods section.

We next sought to quantitate the amount of inflammation observed in EAE rats *in vivo*, with and without prophylactic anti-inflammatory treatment. The total amount of ^19^F signal in the EAE spinal cord and vertebral bone was quantified by normalization to the external reference capillary containing a known amount of ^19^F. In Group 1, we found that an average of 9.5±4.0×10^19^ ^19^F atoms were localized in the spinal cord, whereas 29.0 ±7.1×10^19^ ^19^F atoms were localized in the vertebral bone marrow. In animals receiving prophylactic cyclophosphamide treatment, the ^19^F signal in the spinal cord significantly decreased compared to the untreated EAE spinal cords (Fig 2; 2.6±1.1×10^19^ atoms versus 9.5±4.0×10^19^ atoms, p < 0.05). This ^19^F signal reduction indicates reduced inflammatory burden in the treated animals. Interestingly, the vertebral ^19^F signal was also greatly suppressed in the treatment group when compared to the untreated EAE group (Fig 2; 9.9±2.9×10^19^ atoms versus 29.0±7.1×10^19^ atoms, p < 0.05). Our findings indicate that EAE disease progression induces recruitment of phagocytes to the white matter of the spinal cord and adjacent bone marrow, and the number of recruited phagocytes decreases by approximately 66% after cyclophosphamide treatment (Fig 2).

**Fig 3:**
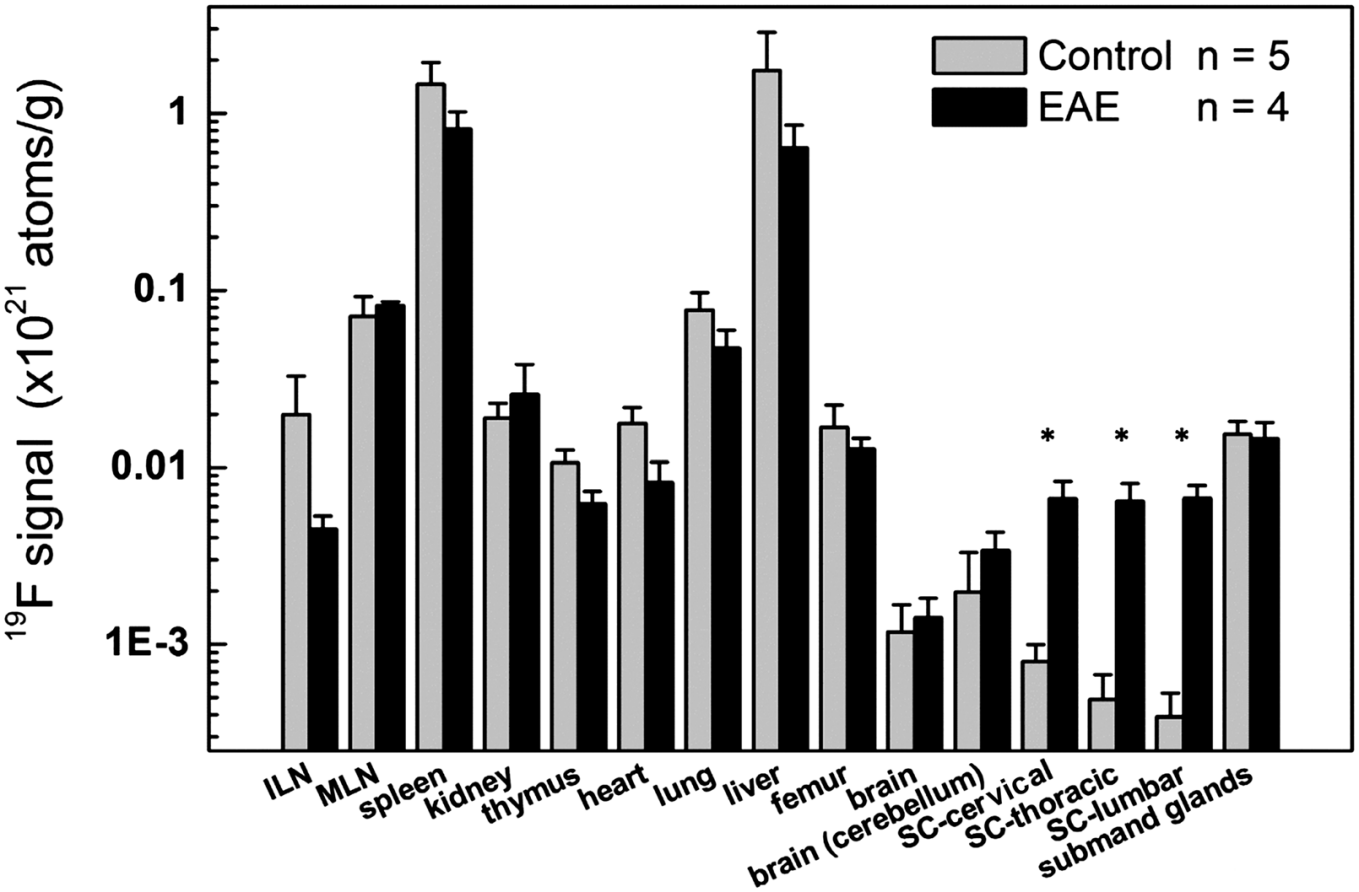
*Ex vivo* ^19^F NMR measurements of organ and tissue biodistribution of the PFC nanoemulsion shows pathological inflammatory infiltrates into the spinal cord of EAE rats. ^19^F signal is significantly higher in the spinal cords of EAE rats compared to wild-type rats. ^19^F signal is high in the liver and spleen of both control and EAE rats because of normal clearance of PFC agent by the RES. (*) indicates p < 0.05 with respect to the control group. ILN = inguinal lymph node, MLN = mesenteric lymph node, and SC = spinal cord. We note that the decrease in ^19^F in the ILN in EAE rates is not statistically significant.

We assayed the biodistribution of the PFC probe to identify possible ‘off-target’ sites of macrophage accumulation outside of the spinal cord and vertebral column in the EAE model. To identify potential inflammatory sites, we used high-resolution NMR instrumentation for ^19^F detection of the remaining intact (fixed) organs and tissues. The *ex vivo* ^19^F NMR results are consistent with the presence of pathologic inflammatory cell infiltrates in the spinal cord and bone marrow in EAE rats (Fig 3). The cervical, thoracic, and lumbar spines all showed significantly higher ^19^F content in EAE rats compared with wild-type rats (p < 0.05, Fig 3). The other organs assayed displayed no significant increase in ^19^F content in EAE rats. As expected, both EAE and control rats showed ^19^F in RES-associated tissues (e.g., liver, spleen, lymph nodes), consistent with the expected clearance route of the PFC nanoemulsion (Fig 3). The average total amount of ^19^F measured by NMR per animal was comparable between the EAE and control rats (4.7±3.0×10^21^ atoms versus 6.3±0.7×10^21^ atoms, respectively, p = NS) two days after PFC administration.

**Fig 4:**
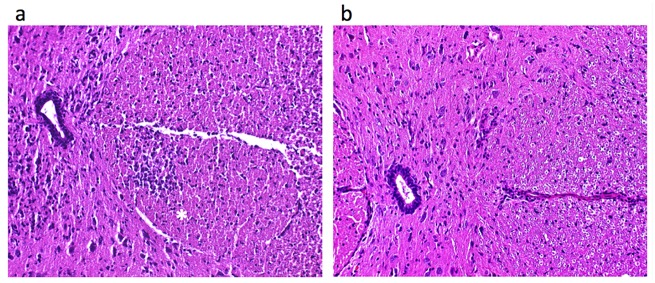
Histolopathologic analyses of spinal cord of EAE rats. H&E stain demonstrates cellular infiltrates (*) in the lateral column of the white matter of the spinal cord of an EAE rat (**a**), without evidence of any corresponding cellular infiltrate in the spinal cord of a control rat (**b**). The central canal of the spinal cord is seen at the left of both images.

Basic histopathologic analyses of rat spinal cord and vertebrae was used to confirm EAE induction. Consistent with MRI results, histology of EAE spinal cords shows pathological inflammatory cell infiltrates in the lateral and dorsal columns of the white matter using H&E stain (Fig 4), which are absent in the control animals. In the exact same EAE model, prior work by Ahrens *et al*. [22] used immunohistochemistry to confirm intracellular co-localization of the PFC droplets within spinal cord CD68+ cells having macrophage morphology.

**Fig 5:**
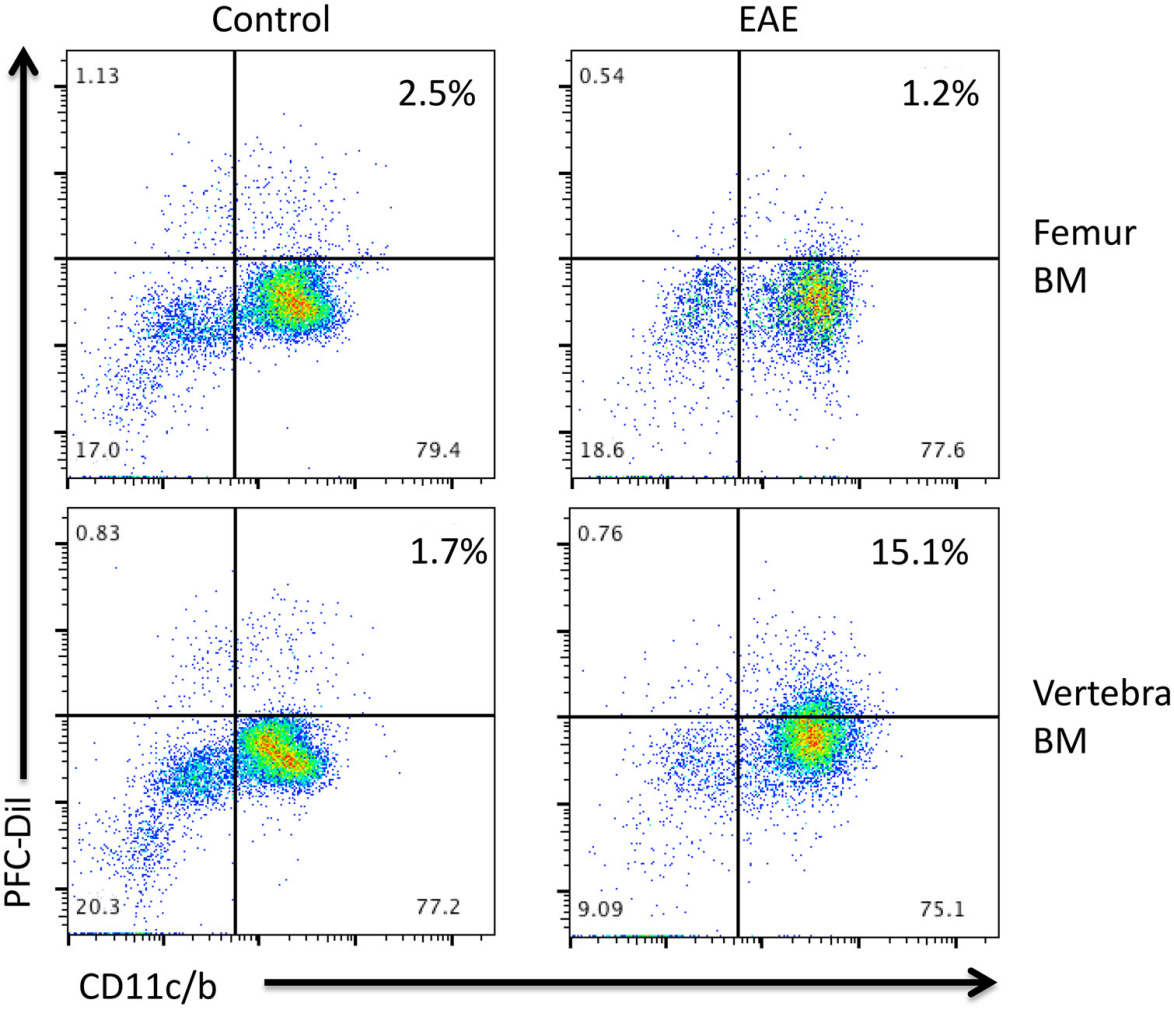
Flow cytometry analysis of bone marrow cells from the femur (top panels) and vertebra (bottom panels). Bone marrow cells from control (left panels) or EAE (right panels) rats were examined for expression of PFC-DiI and CD11c/b on gated CD45^+^ cells. Results shown are representative of two similar experiments.

We also performed preliminary flow cytometry analysis to further identify the cells that were infiltrating the bone marrow sites. In these experiments, EAE or control rats (Groups 6 and 7) were injected with PFC-DiI. Two days later, bone marrow from the femur and vertebral bodies was extracted and analyzed by flow cytometry. The majority of the PFC-DiI positive cells also stained for CD11c/b demonstrating that these cells belong to the myeloid lineage (Fig 5). There was no change in the frequency of PFC-DiI positive myeloid cells in the femoral bone marrow in EAE versus control EAE rats (Fig 5, top panels). However, there was a small increase in PFC-DiI myeloid cells in the vertebral bone marrow of EAE rats (Fig 5, bottom right panel), consistent with the MRI data. The increased accumulation of PFC-DiI positive cells could have been caused by increased migration of myeloid cells to the vertebral bone marrow, or increased proliferation of resident myeloid cells. However, staining for Ki67 did not show an increase in proliferating cells in the vertebral bone marrow (S1 Fig). Future work will determine whether this cellular accumulation is caused by changes in the migration of myeloid cells with additional markers to further delineate the profile of the PFC-DiI positive cells, in addition to cytokine profiling.

## Discussion

In this paper we show that PFC nanoemulsion agents and ^19^F detection can be used to quantitatively assay the spinal cord inflammatory burden of EAE *in vivo*. Surprisingly, we observe that the EAE rats display significant levels of inflammation in the vertebral bone marrow cavities in proximity to the spinal cord lesions, and that these levels are reduced following administration of anti-inflammatory agents. Accumulation of myeloid cells in the vertebral bone marrow in proximity to EAE lesions has not been reported, despite the fact that the disease has been studied since the 1930’s. We speculate that the apparent ^19^F signal observed in the vertebral bone marrow may be caused by up-regulated recruitment and/or production of monocytes/macrophages due to increased cytokine and chemokine concentrations in the vicinity of spinal cord EAE lesions. Previous studies have revealed the importance of infiltrating macrophages in the pathogenesis of EAE [9]. These cells may infiltrate from the adjacent vertebral bone marrow, and the increased presence of PFC-positive macrophages in the vertebral bone marrow may be a marker of increased migration. Preliminary flow cytometry studies did not demonstrate evidence of increased proliferation, as measured by Ki67 staining, in the vertebral bone marrow (S1 Fig).

We note that bone marrow is inherently difficult to analyze unambiguously using conventional ^l^H MRI and metal-ion-based contrast agents. Moreover, alternative molecular imaging techniques, such as bioluminescence imaging [35, 36] and positron emission tomography (PET) [37] have been used to detect EAE inflammation, but these techniques have limited spatial resolution and are challenged to correctly delineate the precise anatomical location of apparent signals within the spinal cord and vertebral column. Also, with PET it can be difficult to differentiate hypermetabolic inflammatory infiltrates from adjacent hypometabolic parenchyma pathology [38].

^19^F MRI offers exceptional specificity because of the extremely low endogenous ^19^F content of tissue, thereby resulting in negligible background signal from the host. Only the ^19^F-labeled cells are detected as hot-spots, and a high ^19^F SNR is not necessary. Quantification of the ^19^F signal is directly proportional to the number of fluorine atoms present in ^19^F spin-density weighted MRI, thereby yielding a quantitative marker of inflammation severity. The detection of PFC labeled cells is intrinsically different from ^1^H MRI contrast agents, where one detects the presence of the contrast agent indirectly via its effect on the relaxation rates of surrounding protons in mobile water. The PFC acts like a ‘tracer’ agent rather than a contrast agent since ^19^F MRI directly detects the ^19^F atoms associated with the labeled cells. The PFC agent used in this study is not degradable *in vivo* by any known enzyme or by acidic pH [39]. The plasma half-life of the PFC nanoemulsion formulation used is ~9.5 hours in rats [21], and it is cleared via the RES system and exhaled through the lungs [39, 40]. Generally, MRI-detectable macrophages labeled with PFC persist in inflammatory lesions for extended periods of time; for example, in a mouse model of inflammatory bowel disease with the same imaging agent [19], macrophages were observed in lesions for at least 30 days after PFC administration. Following systemic administration, PFC nanoemulsions are taken up preferentially by circulating macrophages/monocytes in the blood, but phagocytic engulfment of PFC nanoemulsions has also been demonstrated in very small numbers of B lymphocytes, neutrophils, and dendritic cells [20, 26]. Thus, although the ^19^F signal demonstrated in this study was most likely attributable to circulating phagocytes which had homed to inflammatory loci, it is also possible that other cells contributed to the observed ^19^F signal. In a previous study, we estimated the *in situ* labeling efficiency of the same PFC agent to be approximately 15–20% of all macrophages within the animal [19]. Also, determining the exact PFC uptake per macrophage *in situ* is difficult, unlike for *ex vivo* labeling experiments [32]. Fortunately, the number of ^19^F atoms detected is known to linearly correlate to the number of macrophages and thus the inflammatory burden *in vivo* [19, 22].

Overall, our findings show that *in vivo* ^19^F MRI detection of macrophage burden in the spinal cord and vertebral bone marrow serves as a sensitive quantitative marker for EAE disease burden and the evaluation of treatment strategies.

## Supporting Information

S1 Fig Bone marrow cells from control (left panels) or EAE (right panels) rats were examined for expression of Ki67 and CD11c/b on gated CD45^+^ cells. Results shown are representative of two similar experiments.
(TIFF)

S1 Movie 3D movie of EAE rat MRI. Videos demonstrating volume-rendering of ^19^F MRI signal overlaid upon sagittal ^1^H-MRI of the thoracic spine.
(MP4)

S2 Movie 3D movie of EAE rat MRI. Videos demonstrating volume-rendering of ^19^F MRI signal overlaid upon sagittal ^1^H-MRI of the thoracic spine.
(MP4)

S3 Movie 3D movie of EAE rat MRI. Videos demonstrating volume-rendering of ^19^F MRI signal overlaid upon sagittal ^1^H-MRI of the thoracic spine.
(MP4)
