# Supplementary figures and images for: In Vivo Quantification of Inflammation in Experimental Autoimmune Encephalomyelitis Rats Using Fluorine-19 Magnetic Resonance Imaging Reveals Immune Cell Recruitment outside the Nervous System
Source: PLoS One. 2015 Oct 20;10(10):e0140238. doi: 10.1371/journal.pone.0140238 (PMC4618345; doi:10.1371/journal.pone.0140238)

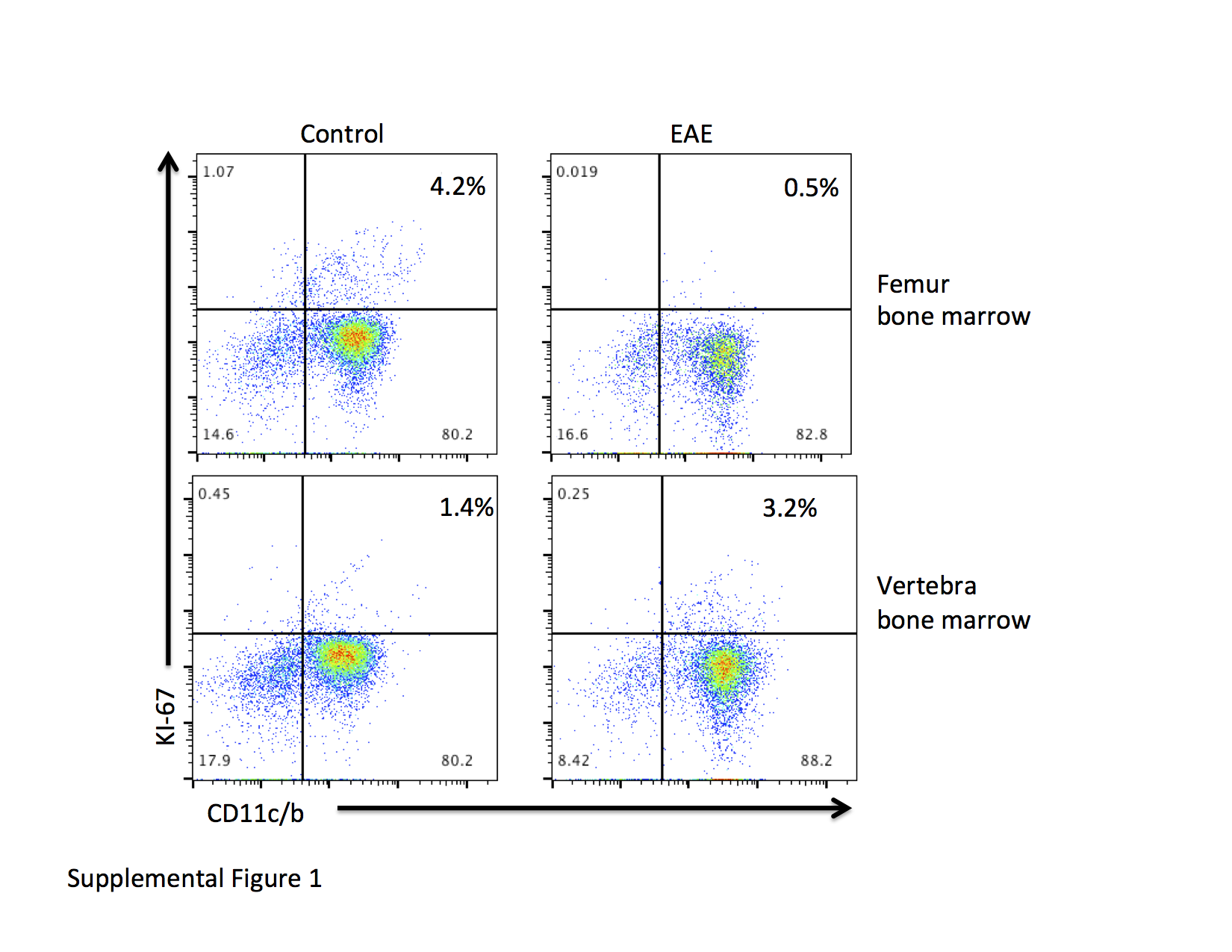

Supplement: S1 Fig — Results shown are representative of two similar experiments. (TIFF) [file pone.0140238.s001.tiff]
